# Supplementary material for: Amplified seasonal cycle in hydroclimate over the Amazon river basin and its plume region
Source: Nat Commun. 2020 Sep 1;11:4390. doi: 10.1038/s41467-020-18187-0 (PMC7463004; doi:10.1038/s41467-020-18187-0)
Supplement: Supplementary file 1 — Supplementary Information [file 41467_2020_18187_MOESM1_ESM.pdf]

Supplementary Information:

Amplified Seasonal Cycle in Hydroclimate over the Amazon River Basin and its Plume Region

Liang et al.

*Correspondence to Yu-Chiao Liang (yliang@whoi.edu)

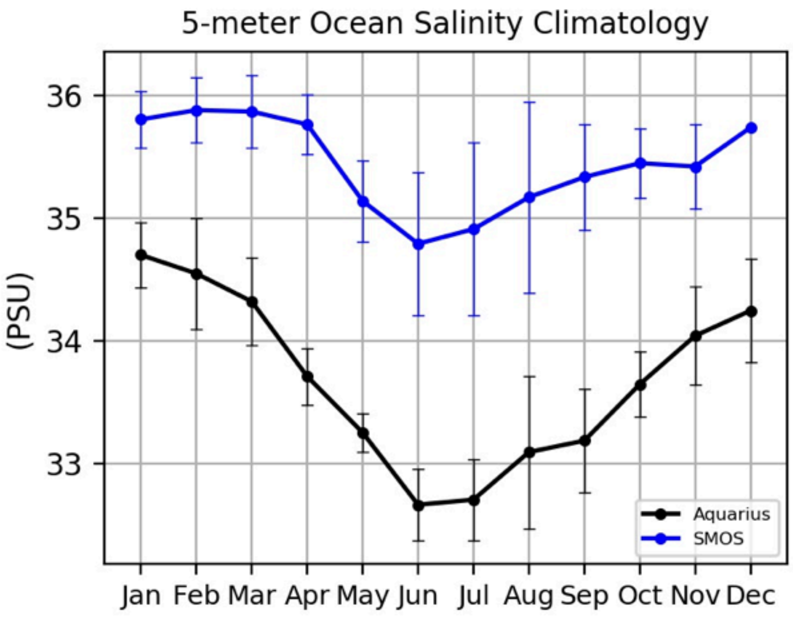

Supplementary Figure 1 | Seasonal cycles of surface or near-surface (5 meter) salinity from satellite observations. The time period of SMOS used in this study is 2011 to 2016, while that of Aquarius is 2012-2014. The error bars denote the standard deviations of each month throughout the respective analysis periods.

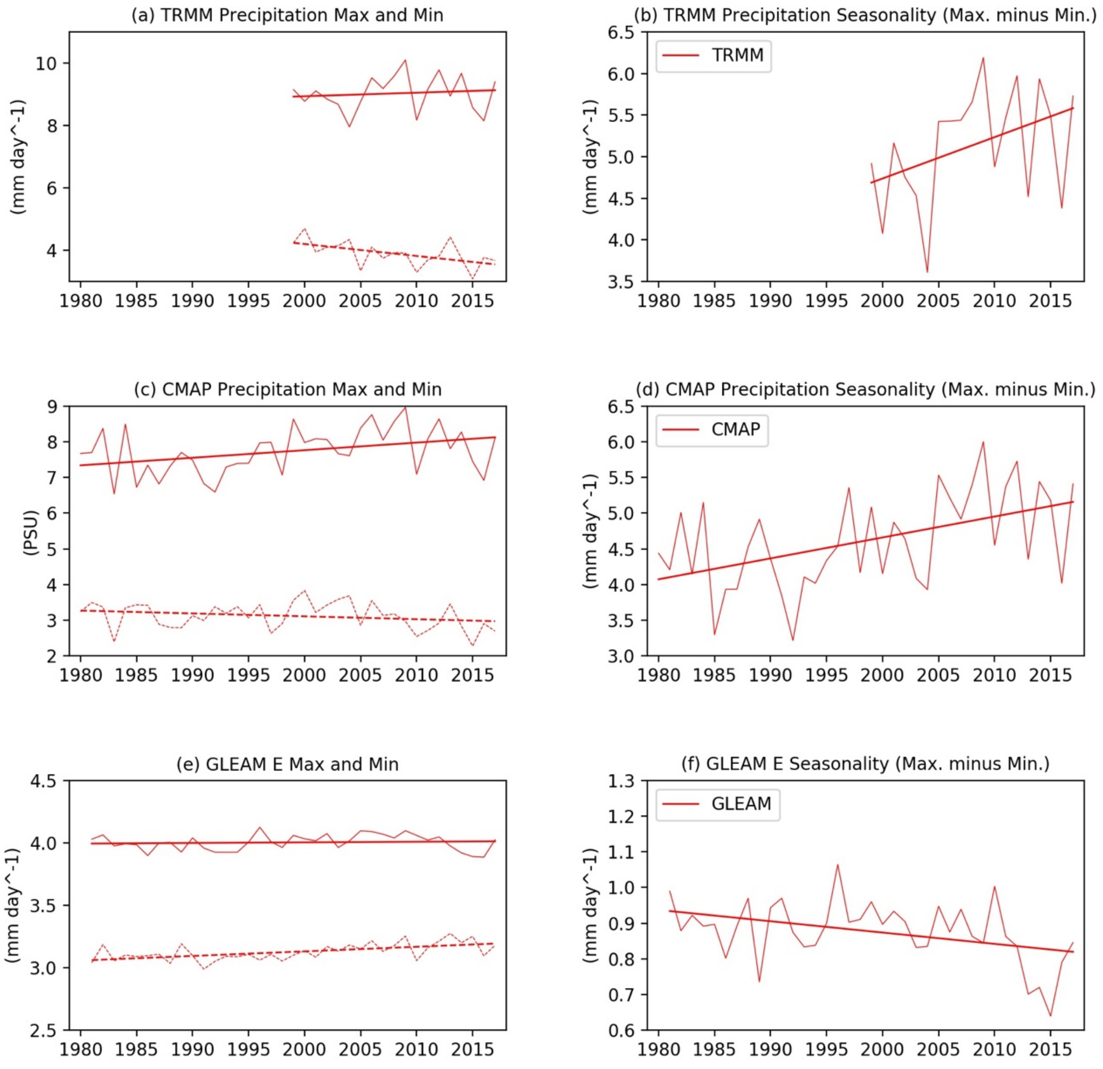

Supplementary Figure 2. | Evolution of maximum and minimum values within one year and seasonality for TRMM and CMAP precipitation and GLEAM Evapotranspiration. a, Maximum and minimum values of TRMM precipitation averaged over the Amazon river basin during the period 1998-2018. **b**, The seasonality (maximum minus minimum values) during the period 1998-2018. The solid (dashed) lines are the linear fit to inform the increasing (decreasing) trends. **c-d** and **e-f** are the same as **a-b** but for CMAP precipitation during 1979-2018 and GLEAM evapotranspiration during 1980-2018 respectively.

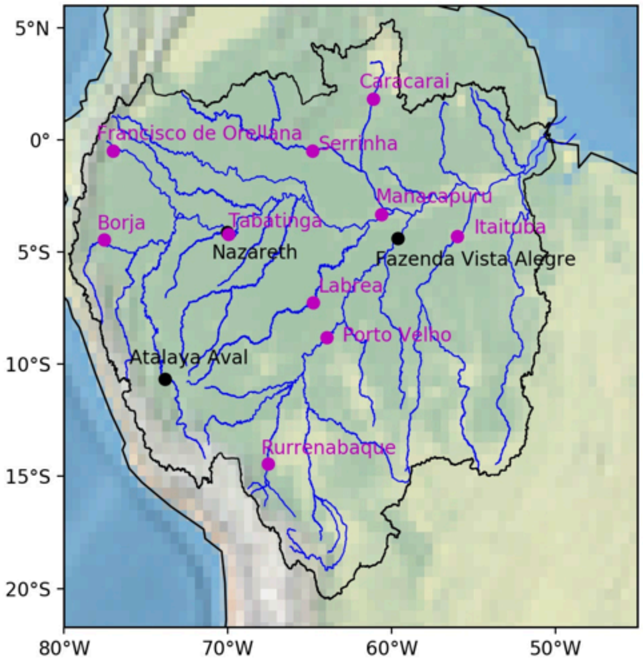

Supplementary Figure 3. | The locations of 13 gauge stations within the Amazon river basin. Magenta locations denote gauge stations with an increasing trend of seasonality, while the black locations denote those with a decreasing trend. The geographic map is produced by Python Cartopy package[1].

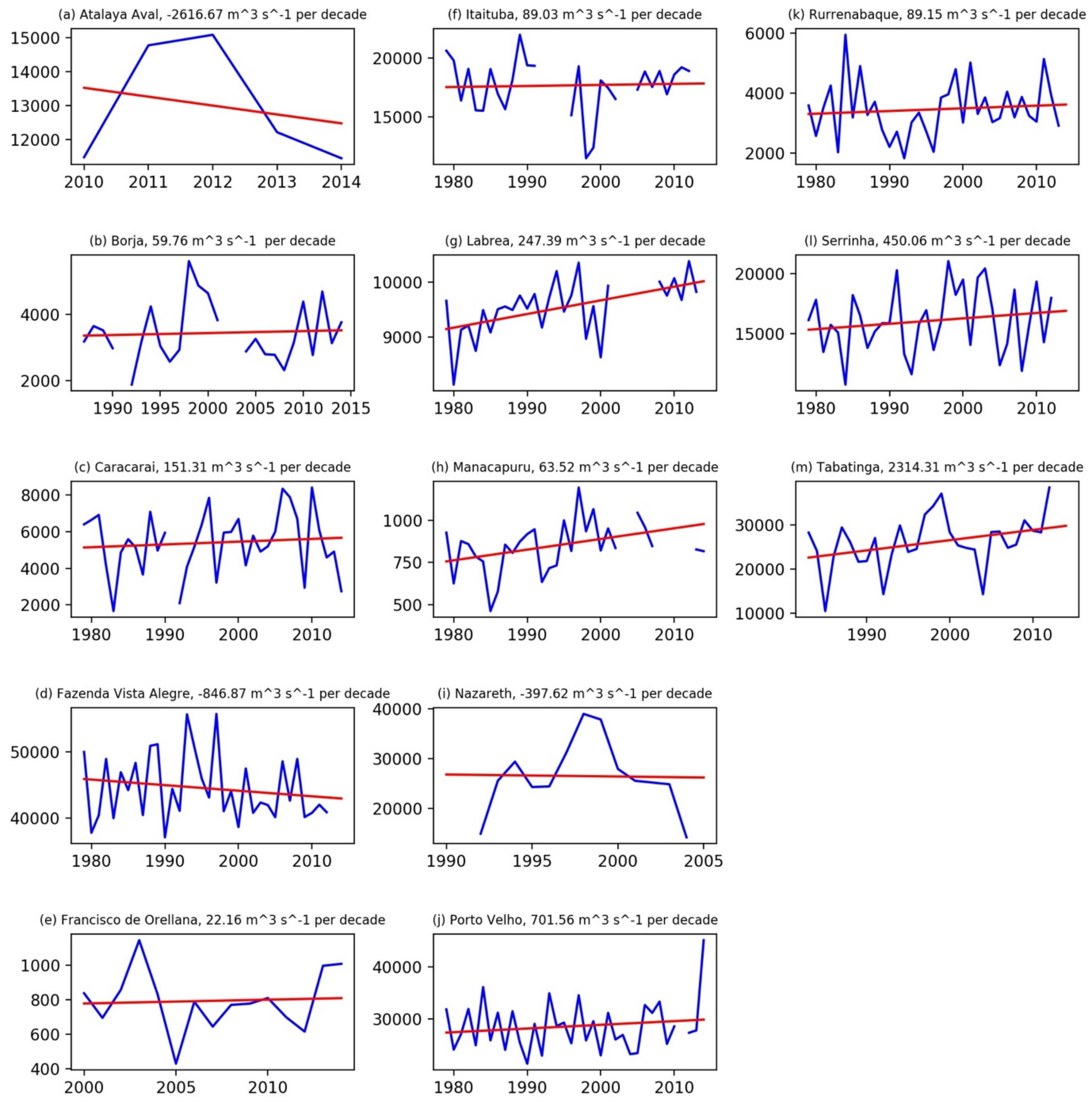

Supplementary Figure 4. | The seasonality of 13 river discharges within the Amazon river basin. a, Atalaya Aval river. **b,** Borja river. **c,** Caracarai river. **d,** Fazenda Vista Alegre river. **e,** Francisco de Orellana river. **f,** Itaituba river. **g,** Labrea river. **h,** Manacapuru river. **i,** Nazareth river. **j,** Porto Velho river. **k,** Rurrenabaque river. **l,** Serrinha river. **m,** Tabatinga river. The red lines indicate the linear trend.

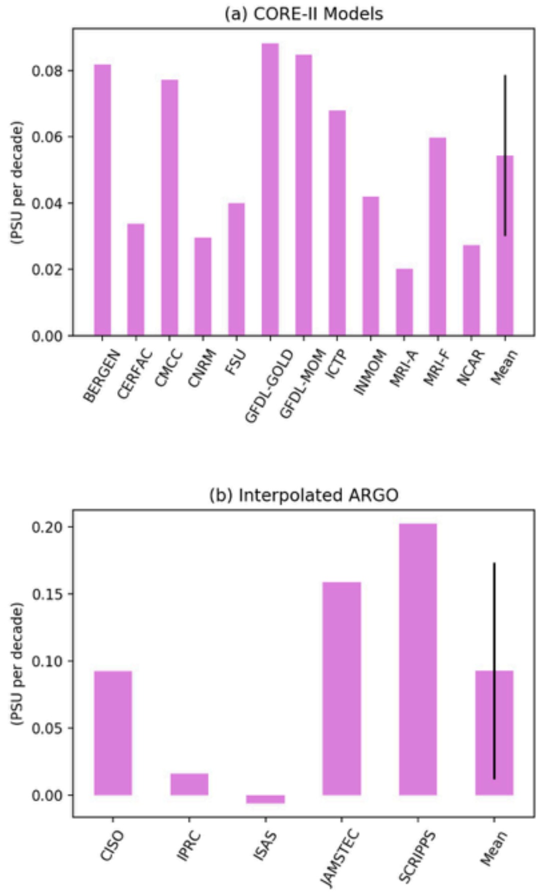

Supplementary Figure 5 | Trends of CORE-II and interpolated ARGO near-surface ocean salinity seasonality. a, Seasonality trends of 12 CORE-II models. **b,** seasonality trends of five interpolated ARGO products. The error bars denote the standard deviations of models and ARGO products.

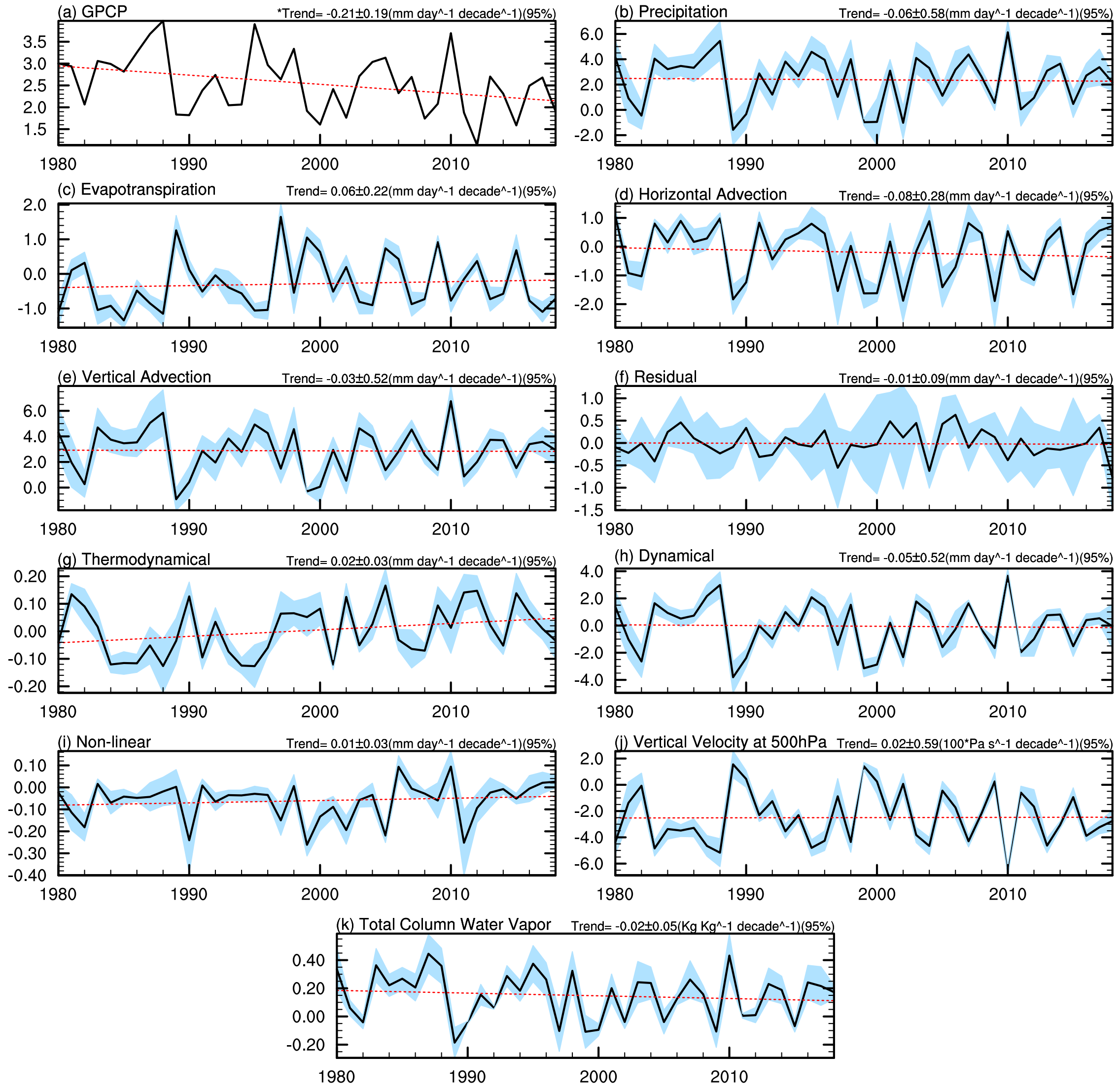

Supplementary Figure 6 | Atmospheric water moisture budget analysis on the seasonality changes during 1979-2018 within APR. The seasonality evolutions of observational precipitation (**a**), reanalysis precipitation (**b**), evapotranspiration (E) (**c**), horizontal moisture advection (-$<\vec{\mathbf{v}} \cdot \nabla q>$) (**d**), vertical moisture advection (-$<\omega \frac{\partial q}{\partial p}>$) (**e**), residual ($\delta$) (**f**), thermodynamic component (-$<\bar{\omega} \frac{\partial q'}{\partial p}>$) (**g**), dynamical component (- $<\omega' \frac{\partial \bar{q}}{\partial p}>$) (**h**), nonlinear component (-$<\omega' \frac{q'}{\partial p}$) (**i**), vertical velocity (ω) (**j**), and total column water vapor (q) (**k**). The blue shadings are the range among reanalysis products used in the calculation and the red lines are the linear fits to inform trends.

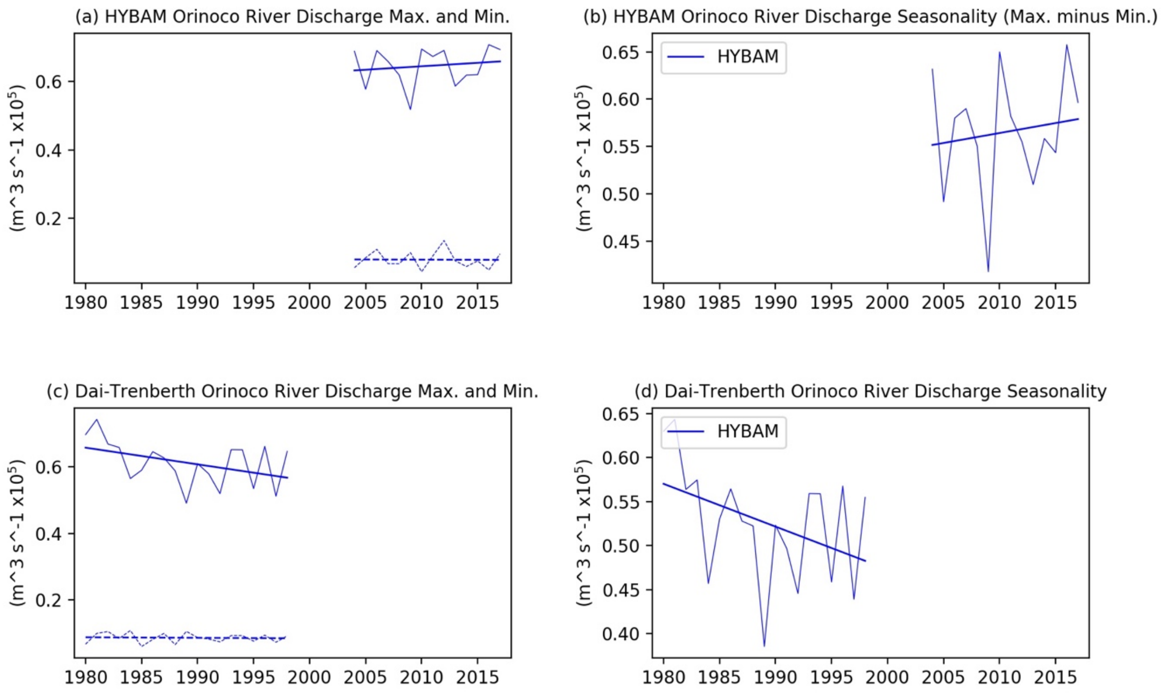

Supplementary Figure 7 | Evolution of maximum and minimum values within one year and seasonality for Orinoco river discharge, and APR ocean salinity. a, Orinoco river discharge maximum and minimum values from HYBAM dataset. **b**, similar to **a** but using Dai-Trenberth dataset. **c-d** are the same as **a-b** but for Dai-Trenberth dataset.

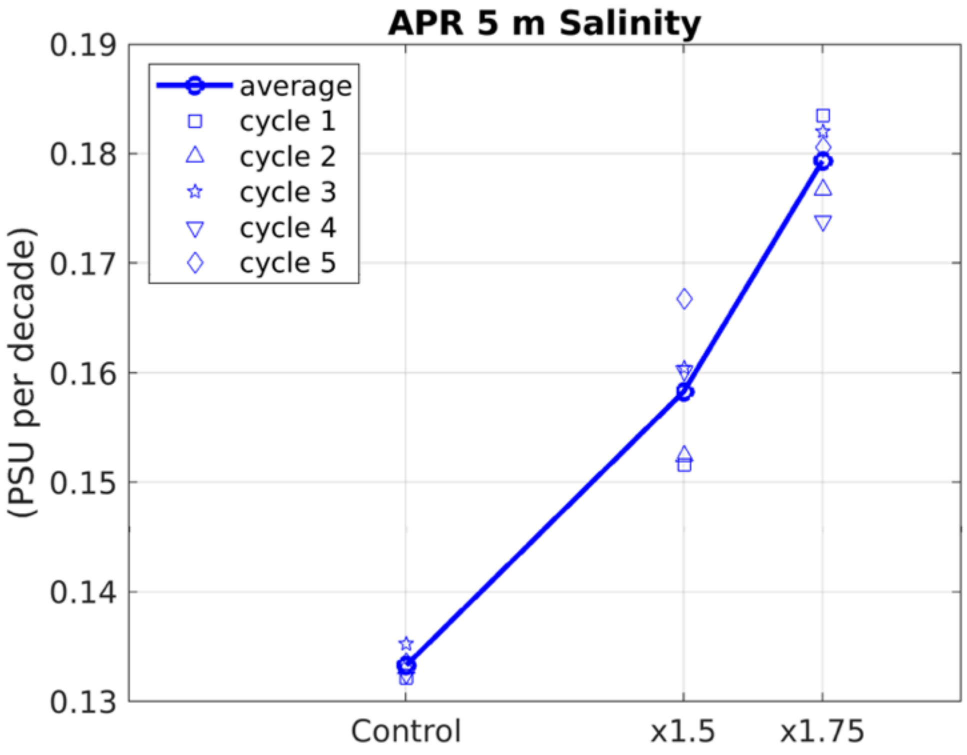

Supplementary Figure 8 | 5-meter ocean salinity seasonality changes with APR defined as regions where salinity is less than 34.5 PSU. Seasonality trends of APR 5-meter ocean salinity seasonality in ocean model experiments.

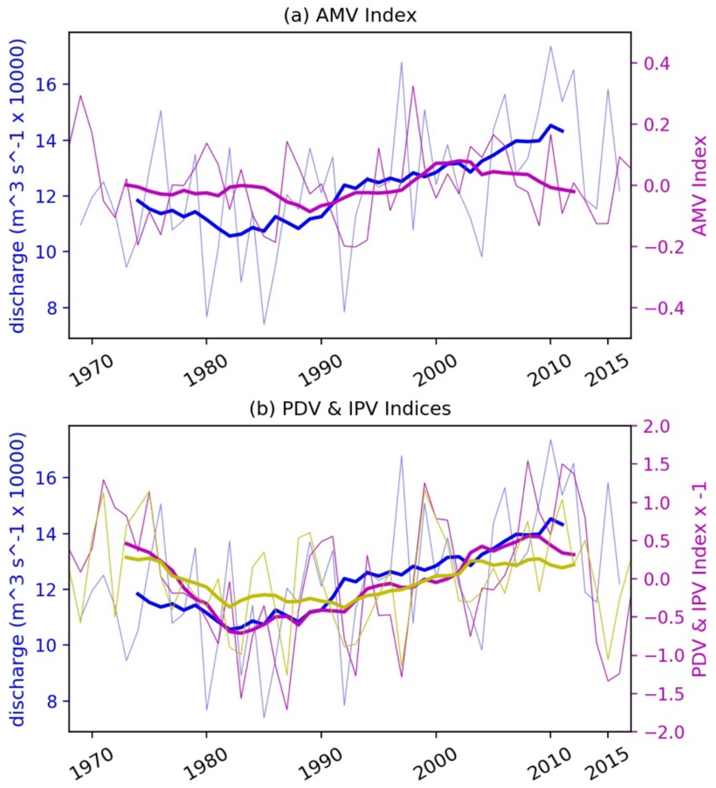

Supplementary Figure 9 | Natural variability. Comparisons for Amazon river discharge with AMV index (magenta line in **a**), PDV index (magenta line in **b**), and IPV index (yellow line in **b**). The blue line represents the seasonality of Amazon river discharge. The thick lines denote the 11-year running average time series, while the thin lines the original time series.

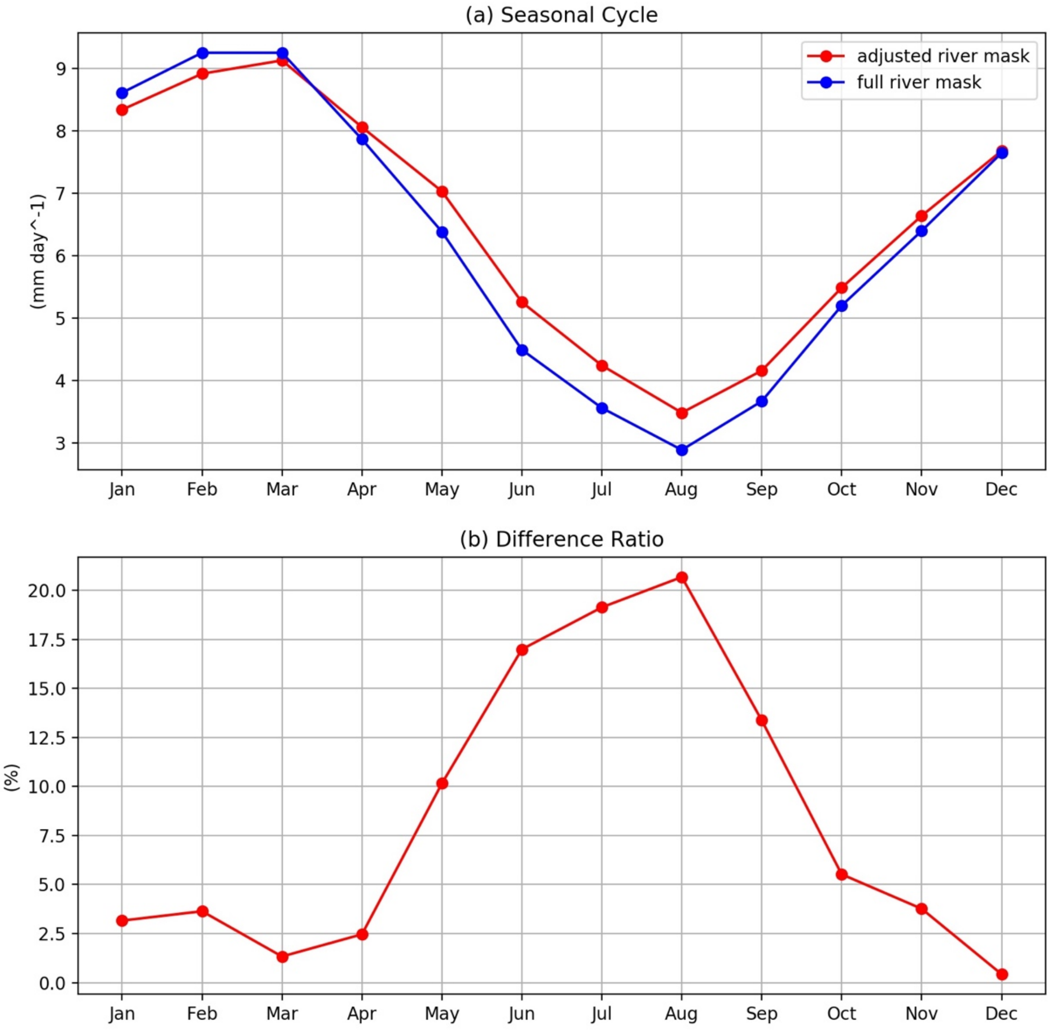

Supplementary Figure 10. | Comparison of adjusted and conventional Amazon river basin. **a**, the seasonal evolution of TRMM precipitation averaged over the adjusted Amazon river basin (red line) and conventional one (blue line). **b**, the absolute difference between the two as a percentage.

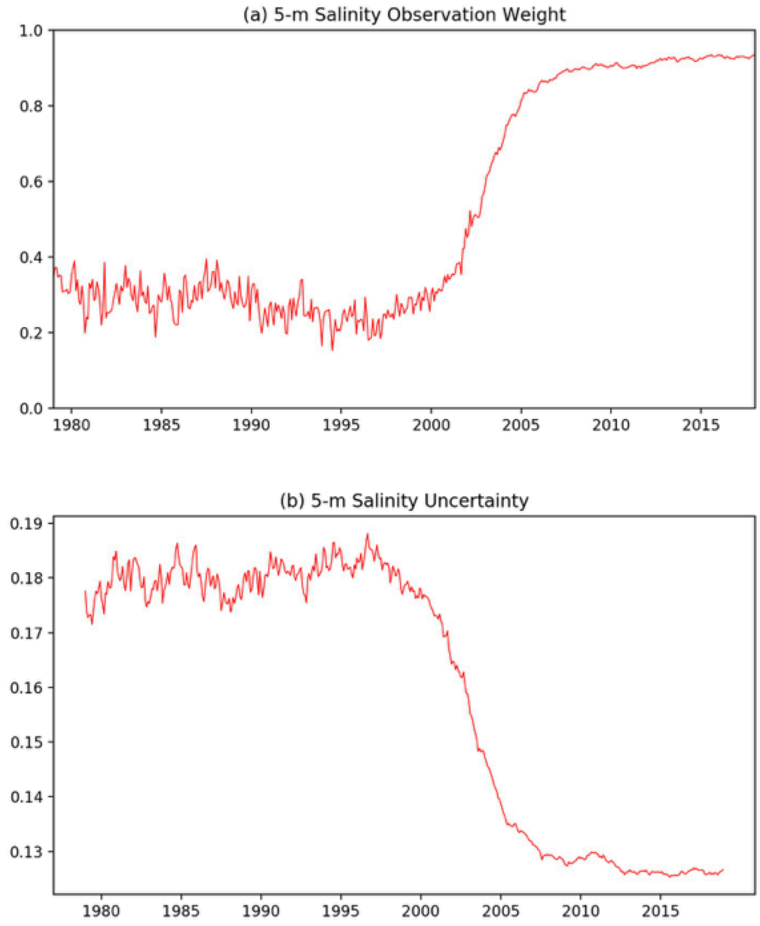

Supplementary Figure 11. | EN4 5-meter salinity data quality in the APR. **a**, The salinity observation weight averaged over the APR. **b**, similar to **a** but for salinity uncertainty.

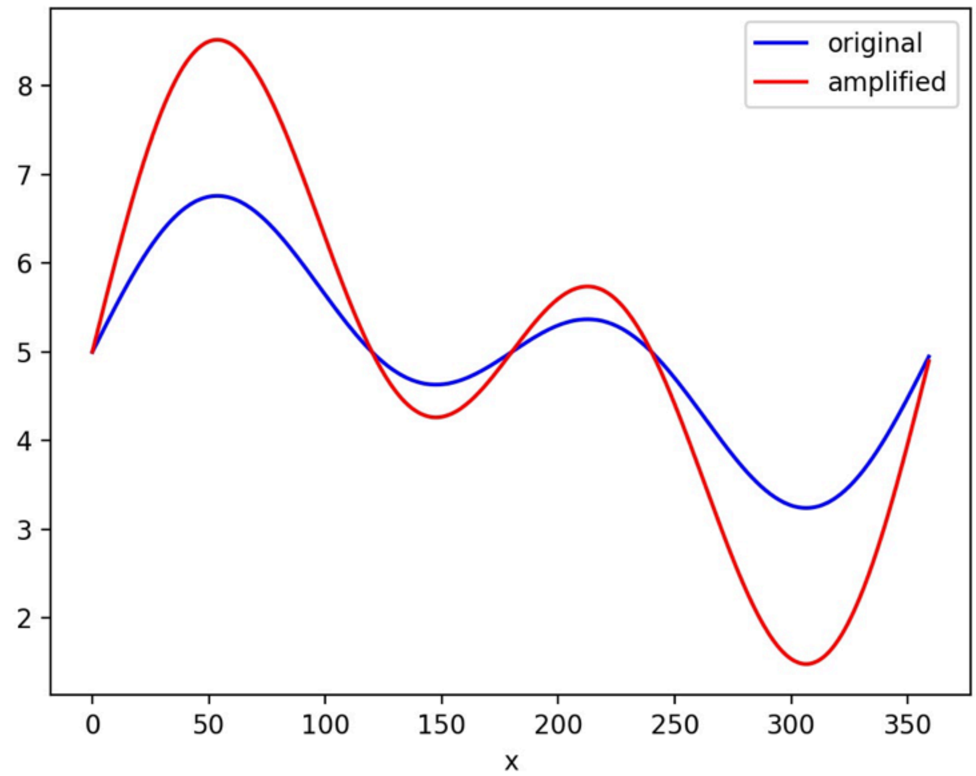

Supplementary Figure 12 | Idealised wave amplitude enhancement. The blue line is a combined sine wave $\sin(\frac{2\pi}{360}x) + \sin\left(\frac{4\pi}{360}x\right) + 5$, while the red line has its amplitude increased by a factor of 2.

Supplementary Table 1. Precipitation trends in mm day^-1 per decade (bold number: 5% significance)

Dataset	Period	Max. Trend	Min. Trend	Max. - Min. (ΔS) Trend
GPCP	1980-2017	**2.43×10^{-1}**	**-1.49×10^{-1}**	**3.91×10^{-1}**
GPCC	1980-2017	**1.70×10^{-1}**	**-1.07×10^{-1}**	**2.76×10^{-1}**
PREC/L	1980-2017	**2.24×10^{-1}**	**-1.50×10^{-1}**	**3.74×10^{-1}**
ERA5[2]	1980-2017	**2.21×10^{-1}**	**-2.47×10^{-1}**	**4.68×10^{-1}**
ERAI[3]	1980-2017	**6.52×10^{-1}**	**2.70×10^{-1}**	**3.82×10^{-1}**
JRA55[4]	1980-2017	**4.13×10^{-1}**	**1.77×10^{-1}**	**2.36×10^{-1}**
NCEP_R1[5]	1980-2017	**7.81×10^{-1}**	**3.77×10^{-1}**	**4.05×10^{-1}**
NCEP_R2[6]	1980-2017	**4.71×10^{-1}**	1.79×10^{-1}	2.92×10^{-1}
Multi-Mean Reanalysis	1980-2017	**5.08×10^{-1}**	**1.51×10^{-1}**	**3.56×10^{-1}**

Supplementary Table 2. Amazon river discharge trends in m^3 s^-1 per decade (bold number: 5% significance)

Dataset	Period	Max. Trend	Min. Trend	Max. - Min. (ΔS) Trend
HYBAM	1980-2017	**9.97×10^{3}**	-2.76×10^{3}	**1.27×10^{4}**
Dai & Trenberth*	1980-2007	**1.09×10^{4}**	**-2.32×10^{3}**	**1.32×10^{4}**

*missing Amazon river discharge data after 2007 in Dai & Trenberth

Supplementary Table 3. Ocean salinity trends in PSU per decade (bold number: 5% significance)

Dataset	Period	Max. Trend	Min. Trend	Max. - Min. (ΔS) Trend
GECCO2	1980-2015	1.76×10^{-2}	-1.13×10^{-2}	**2.89×10^{-2}**
ECCO4	1993-2016	-3.68×10^{-3}	-1.45×10^{-2}	1.82×10^{-2}
ORAS5	1980-2017	1.43×10^{-2}	4.12×10^{-2}	-2.72×10^{-2}
SODA3.3.1	1981-2014	-8.81×10^{-3}	**-1.52×10^{-1}**	**1.23×10^{-1}**
EN4	1980-2013	4.38×10^{-2}	1.14×10^{-1}	-7.04×10^{-2}

Supplementary Table 4. Evapotranspiration trends in mm day^-1 per decade (bold number: 5% significance)

Dataset	Period	Max. Trend	Min. Trend	Max. - Min. (ΔS) Trend
GLEAM	1981-2017	-1.71×10^{-1}	**1.12**	**-9.52×10^{-1}**
ERA5	1980-2017	-8.88×10^{-4}	4.65×10^{-3}	-5.50×10^{-3}
ERAI	1980-2017	**-5.29×10^{-2}**	**6.16×10^{-2}**	**-1.14×10^{-1}**
JRA55	1980-2017	**-1.47×10^{-1}**	**7.10×10^{-2}**	**-2.18×10^{-1}**
NCEP_R1	1980-2017	**-2.04×10^{-1}**	-3.84×10^{-3}	**-2.00×10^{-1}**
NCEP_R2	1980-2017	**-1.04×10^{-1}**	**1.15×10^{-1}**	**-2.20×10^{-1}**
Multi-Mean Reanalysis	1980-2017	**-1.02×10^{-1}**	**4.97×10^{-2}**	**-1.52×10^{-1}**

Supplementary Table 5. Horizontal moisture advection trends in mm day^-1 per decade (bold number: 5% significance)

Dataset	Period	Max. Trend	Min. Trend	Max. - Min. (ΔS) Trend
ERA5	1980-2017	$3.63x10^{-2}$	**$1.86x10^{-1}$**	**$-6.90x10^{-2}$**
ERAI	1980-2017	**$-6.66x10^{-2}$**	**7.02 $x10^{-3}$**	**$-2.79x10^{-1}$**
JRA55	1980-2017	$-7.59x10^{-3}$	**$5.97x10^{-1}$**	**$-1.72x10^{-1}$**
NCEP_R1	1980-2017	**$-9.30x10^{-2}$**	**$3.27x10^{-3}$**	**$-2.20x10^{-1}$**
NCEP_R2	1980-2017	**$-6.15x10^{-2}$**	**$2.10x10^{-2}$**	**$-2.70x10^{-1}$**
Multi-Mean Reanalysis	1980-2017	**$-3.85x10^{-2}$**	**$4.59x10^{-2}$**	**$-2.02x10^{-1}$**

Supplementary Table 6. Vertical moisture advection trends mm day^-1 per decade (bold number: 5% significance)

Dataset	Period	Max. Trend	Min. Trend	Max. - Min. (ΔS) Trend
ERA5	1980-2017	**$1.61x10^{-1}$**	**$-1.52x10^{-1}$**	**$3.13x10^{-1}$**
ERAI	1980-2017	**$9.02x10^{-1}$**	**$4.23x10^{-1}$**	**$4.79x10^{-1}$**
JRA55	1980-2017	**$3.86x10^{-1}$**	**$1.42x10^{-1}$**	**$2.45x10^{-1}$**
NCEP_R1	1980-2017	**1.07**	**$2.25x10^{-1}$**	**$8.42x10^{-1}$**
NCEP_R2	1980-2017	**$8.60x10^{-1}$**	$1.35x10^{-1}$	**$7.24x10^{-1}$**
Multi-Mean Reanalysis	1980-2017	**$6.75x10^{-1}$**	**$1.55x10^{-1}$**	**$5.21x10^{-1}$**

Supplementary Table 7. Thermodynamic Component trends mm day^-1 per decade (bold number: 5% significance)

Dataset	Period	Max. Trend	Min. Trend	Max. - Min. (ΔS) Trend
ERA5	1980-2017	$3.32x10^{-2}$	**$-1.96x10^{-2}$**	**$5.27x10^{-2}$**
ERAI	1980-2017	**$7.52x10^{-2}$**	**$2.45x10^{-2}$**	**$5.07x10^{-2}$**
JRA55	1980-2017	$3.36x10^{-2}$	$3.10x10^{-3}$	$3.05x10^{-2}$
NCEP_R1	1980-2017	**$1.68x10^{-1}$**	**$3.43x10^{-2}$**	**$1.34x10^{-1}$**
NCEP_R2	1980-2017	**$7.66x10^{-2}$**	**$2.45x10^{-3}$**	**$7.42x10^{-2}$**
Multi-Mean Reanalysis	1980-2017	**$7.73x10^{-2}$**	$8.96x10^{-3}$	**$6.83x10^{-2}$**

Supplementary Table 8. Dynamic Component trends mm day^-1 per decade (bold number: 5% significance)

Dataset	Period	Max. Trend	Min. Trend	Max. - Min. (ΔS) Trend
ERA5	1980-2017	$1.34x10^{-1}$	**$-1.30x10^{-1}$**	**$2.64x10^{-1}$**
ERAI	1980-2017	**$8.19x10^{-1}$**	**$3.84x10^{-1}$**	**$4.35x10^{-1}$**
JRA55	1980-2017	**$3.48x10^{-1}$**	**$1.34x10^{-1}$**	**$2.14x10^{-1}$**
NCEP_R1	1980-2017	**$8.77x10^{-1}$**	**$1.74x10^{-1}$**	**$7.04x10^{-1}$**
NCEP_R2	1980-2017	**$7.75x10^{-1}$**	$1.21x10^{-1}$	**$6.55x10^{-1}$**
Multi-Mean Reanalysis	1980-2017	**$5.91x10^{-1}$**	**$1.37x10^{-1}$**	**$4.54x10^{-1}$**

References

1. Met Office. Cartopy: a carographic python library with a Matplotlib interface. Exeter, Devon (2010-2015), https://scitools.org.ul/cartopy.
2. 2. Dee, D. P., et al. The ERA-Interim reanalysis: Configuration and performance of the data assimilation system. *Q. J. R. Meteorol. Soc.* **137**, 553-597 (2011).
3. Hersbach, H., et al. Global reanalysis: goodbye ERA Interim, hello ERA5. *ECMWF News* **159**, 17-24 (2019).
4. Kobayashi, S., et al. The JRA-55 reanalysis: General specifications and basic characteristics. *J. Meteorol. Soc. Jpn.* **1**, 5-48 (2015).
5. Kalnay et al. The NCEP/NCAR 40-Year Reanalysis Project. *Bull. Amer. Meteor. Soc.* **77**, 437-471 (1996).
6. Kanamitsu, M., et al. NCEP-DOE AMIP-II Reanalysis (R-2). *Bull. Amer. Meteor. Soc*. **83**, 1631-1643 (2002).
